# A Noise-Filtering Method for Link Prediction in Complex Networks

**DOI:** 10.1371/journal.pone.0146925

**Published:** 2016-01-20

**Authors:** Bo Ouyang, Lurong Jiang, Zhaosheng Teng

**Affiliations:** 1 College of Electrical and Information Engineering, Hunan University, Changsha, Hunan Province, China; 2 School of Information Science and Technology, Zhejiang Sci-Tech University, Hangzhou, Zhejiang Province, China; Beihang University, CHINA

## Abstract

Link prediction plays an important role in both finding missing links in networked systems and complementing our understanding of the evolution of networks. Much attention from the network science community are paid to figure out how to efficiently predict the missing/future links based on the observed topology. Real-world information always contain noise, which is also the case in an observed network. This problem is rarely considered in existing methods. In this paper, we treat the existence of observed links as known information. By filtering out noises in this information, the underlying regularity of the connection information is retrieved and then used to predict missing or future links. Experiments on various empirical networks show that our method performs noticeably better than baseline algorithms.

## Introduction

About one and a half decades ago, Barabási and Albert pointed out that the property of scale-invariance of many real networked systems originates from a specific growth process, named preferential attachment [1]. Since then, the study of complex networks has led to dramatic changes in many different fields [2–7], and also, many facets of node attractiveness in growing networks, rather than preferential attachment, have been revealed, e.g. similarity [8]. Since different growing processes often result in networks with strikingly different macroscopic properties, how real-world networks are evolved is a fundamental question in understanding our complex world. Link prediction, one of whose capabilities is to rank the best candidates of future links, plays an important role in revealing the evolution processes of networks [9, 10].

On the other hand, many applications have to predict missing links in networked systems [11–13]. Determining whether a link exists in such networks is usually very costly, yet the answer is crucial. For example, knowing the map of protein-protein interactions will reveal many aspects of the cellular function [14], but little has been studied. Link prediction are also widely used in these applications [15, 16].

The problem of link prediction has received much attention from the network science community in the past few years [9, 12, 17, 18]. In general, both topological feature and node attributes can be used in the prediction. However, the latter is usually unavailable or unreliable. For example, in online social networks, the personal information of users are inaccessible due to privacy policies. Thus, many algorithms consider only topological features.

Basically, there are two classes of topological methods—similarity-based and likelihood-based algorithms. Similarity based algorithms assume that two nodes are likely to be connected if they are similar. It assigns a score *s*_*xy*_ to each pair of nodes *x* and *y*, which is defined as the similarity between them. All non-observed links are ranked according to their scores, and the links connecting more similar nodes are supposed to be of higher existence likelihoods. A wealth of methods of this type have been proposed. For example, CN (Common Neighbours) [19] uses the number of common neighbours to rank the similarity of nodes and the likelihood that they are/will be linked. Many variations of CN are also proposed: AA (Adamic-Adar) [20], Resource Allocation (RA) [19] give more importance to common neighbours with lower degree, and Jaccard’s index is a normalised CN. Only local structural information are used in these methods. There are also methods utilizing quasi-global or global information. For example, the Local Path method defines the similarity as the number of paths passing through two nodes, whose length may be larger than 2.

Recently, the organization patterns existing in many real-world networks are utilized in predicting missing links. Likelihood-based methods make assumptions of the structure, with specific parameters obtained by maximising the likelihood of the known structure. Predictions of the non-observed links are made based on the presumed pattern and the parameters. For example, Ref. [21] utilizes the hierarchical structure existing in many networks to predict missing links. And Cannistraci et al. propose the local-community-paradigm to improve the performance of classical predictors [13].

We know that real-world information always contains noise, which is also the case in an observed network. However, this problem is rarely considered in existing methods. In Ref. [18], the authors use the average of the eigen-decomposition of perturbed adjacency matrix (by removing some links) to suppress the noise. However, the underlying physical meaning is not clear, say, why should the eigenvectors of the adjacency matrix reflect the regularity of a network, if they actually are sensitive to perturbation [22]? Besides, it has a high computational complexity. In this paper, by treating the existence of observed links as known “information” (as in [23, 24]), and filtering out the noise in it, we obtain similarity scores for all non-observed links. We give a more theoretical analysis of the link prediction problem and a more meaningful demonstration of the noise-filtering (NF) method. Our method outperforms the typical predictors.

## Materials and Methods

### Metrics

In this paper, two metrics are used to compare the performance of the base-line algorithms and the proposed noise-filtering method.

Consider that we are given an simple network *G*(*V*, *E*), where *V* and *E* are the set of nodes and links, respectively. By “simple”, we mean there are no self-loops or multi-links in the network. In a similarity-based algorithm, for each pair of nodes *x*, *y* ∈ *V* without a link, a similarity score is assigned. Then all unlinked pairs are ranked in descending order according to their scores, and the links on the top are considered as the ones with the highest likelihoods to be connected.

To test the accuracy of a predictor, we randomly divide the observed links in the network into a training set *E*^*T*^ and a probe set *E*^*P*^. Here, *E*^*T*^ is treated as known information while *E*^*P*^ is only used to test the accuracy. Clearly, we have *E*^*T*^∪*E*^*P*^ = *E* and *E*^*T*^∩*E*^*P*^ = ∅.

In this study, we use two metrics, AUC (Area Under the Receiver operating characteristic curve) and precision to evaluate the performance of a predictor. They are defined as follows.

- AUC: AUC is a metric in the receiver operating characteristics (ROC) analysis [25]. Taking the top *L* links as predicted links, a ROC curve is obtained by plotting true positive rates versus false positive rates for varying *L* values. Thus AUC can be interpreted as the probability that a randomly chosen missing link (i.e., a link in *E*^*P*^) has a higher score than a randomly chosen non-existent link (i.e., a link in *U* − *E*), in the rank of all non-observed links. In the algorithmic implementation, if among *n* times of independent comparisons, there are *n*′ times in which the score of the missing link is higher than that of the non-existent link and *n*′′ times in which the two have the same score, then AUC can be expressed as
  $$\begin{array}{c} AUC=\frac{n^{\prime }+n^{\prime \prime }}{n}\;. \end{array}$$ (1)
  If all the scores are generated from an independent and identical distribution, AUC will be approximately 0.5. Therefore, the extent to which AUC exceeds 0.5 indicates how much better the algorithm performs than pure chance.
- Precision: Given the ranking of the non-observed links, the precision is defined as the ratio of relevant items selected to the number of items selected. Thus if we choose the top-*L* links in the rank, and there are *L*_*r*_ links correctly predicted, then
  $$\begin{array}{c} Precision=L_{r}/L\;. \end{array}$$ (2)
  Clearly, higher precision means higher accuracy. In this paper, *L* is always set to the size of the probe set.

### Data Description

Networks from different fields are considered in the experiment, including biological, social, and technological networks. The original networks are turned into undirected, and simple (with multiple links or loops removed) networks. These networks are described in the following. i) Karate [26]: A social network of a university karate club. ii) FoodWeb [27]: A food web in Florida Bay during the rainy season. iii) Jazz [28]: A collaboration network of jazz musicians. iv) Neural [29]: The neural network of C.elegans. v) USAir [30]: The US Air transportation network. vi) Metabolic: The metabolic network of C.elegans. vii) Email [31]: A network of Alex Arenas’s email. viii) PB [32]: A network of US political blogs. ix) Yeast [33]: A protein-protein interaction network. x) EPA [34]: A network of web pages linking to the website www.epa.gov.xi) Router [35]: The router-level topology of the Internet. xii) WikiVote [36, 37]: The network contains all the Wikipedia voting data from its inception till January 2008. Their basic topological parameters are summarized in Table 1.

**Table 1:** Topological parameters of the real-world networks.

	|*V*|	|*E*|	*C*	*r*	〈*k*〉	*H*
Karate	34	78	0.571	-0.476	4.588	1.693
FoodWeb	128	2075	0.335	-0.112	32.422	1.237
Jazz	198	2742	0.617	0.020	27.697	1.395
Neural	297	2148	0.292	-0.163	14.465	1.801
USAir	332	2126	0.625	-0.208	12.807	3.464
Metabolic	453	2025	0.646	-0.226	8.940	4.485
Email	1133	5451	0.220	0.078	9.622	1.942
PB	1490	16715	0.263	-0.221	22.436	3.622
Yeast	2361	6646	0.130	-0.099	5.630	2.944
EPA	4772	8909	0.064	-0.303	3.734	7.573
Router	5022	6258	0.012	-0.138	2.492	5.503
WikiVote	8297	100762	0.121	-0.083	24.289	5.985

|*V*| and |*E*| are the number of nodes and links. *C* is the clustering coefficient and *r* the degree-degree correlation coefficient. 〈*k*〉 is the average degree, 〈*d*〉 is the average shortest distance, and *H* is the degree heterogeneity *H* = 〈*k*^2^〉/〈*k*〉^2^.

### Baseline Algorithms for Comparison

In this paper, six representative similarity indices are considered for performance comparison, including the Common Neighbours (CN), Adamic-Adar (AA) [20], Resource Allocation (RA) [19], Preferential Attachment (PA) [38], Local Path (LP) [39], and Katz [40]. The first four are local indices, the fifth is a quasi-local index, and the last is a global index. Some of them are briefly introduced earlier. Here we present the details of these algorithms.

1. CN index. The CN index follows the intuition that two nodes *x* and *y* are more likely to have connection if their nearest neighbours overlap substantially. The similarity score is obtained by
  $$\begin{array}{c} s_{xy}=\left|\Gamma \left(x\right)\cap \Gamma \left(y\right)\right|\;, \end{array}$$ (3)
  where Γ(*x*) is the set of neighbours of *x* and | ⋅ | denotes the cardinality of a set.
2. AA index. AA is a variation of CN: it gives less importance to common neighbours with high degree:
  $$\begin{array}{c} s_{xy}=\sum_{s\in \Gamma (x)\cap \Gamma (y)}\frac{1}{\log|\Gamma (s)|}\;. \end{array}$$ (4)
3. RA index. Similar to AA, the only difference is that RA punishes high-degree common neighbours to a higher extent:
  $$\begin{array}{c} s_{xy}=\sum_{s\in \Gamma (x)\cap \Gamma (y)}\frac{1}{\left|\Gamma (s)\right|}\;. \end{array}$$ (5)
4. PA index. The PA index supposes that popular nodes are more likely to be connected to. This index is defined as
  $$\begin{array}{c} s_{xy}=|\Gamma \left(x\right)||\Gamma \left(y\right)|\;. \end{array}$$ (6)
5. LP index. Unlike the previous indices, LP uses second order information (information about neighbours of the neighbours) to improve performance. It is defined by
  $$\begin{array}{c} s_{xy}={\left(A^{2}\right)}_{xy}+\epsilon {\left(A^{2}\right)}_{xy}\;. \end{array}$$ (7)
6. Katz index. This index sums over the number of paths (including loops) between two nodes, with each number exponentially damped by the path length
  $$\begin{array}{c} s_{xy}=\beta A_{xy}+\beta^{2}{\left(A^{2}\right)}_{xy}+\beta^{3}{\left(A^{3}\right)}_{xy}+\cdots ={\left(I-\beta A\right)}^{-1}-I\;. \end{array}$$ (8)

Note that the LP index and Katz are both parameter-dependent.

## Results

### Link Prediction via Noise Filtering

In many networks, the formation of links usually embodies both regularities and irregularities. Only the former shows a uniform pattern, which is called the intrinsic pattern. For a specific link, if its existence does not correspond with this pattern, then its existence should be treated as noise. For a specific link, if its existence does not correspond with the connection pattern of the whole network, then its existence is treated as noise. A large body of link prediction methods (i.e. common neighbor method) assumes that nodes are linked if they are similar. Following this assumption, we treat links connecting dissimilar nodes as noise. By filtering out the noise, we can obtain the intrinsic connection pattern, which can be further used to predict missing or future links.

To this end, one has to define a measure to quantify the degree to which a link connects dissimilar nodes.

For every node in the network, assume that its topological features are captured by some vectors in $R^{m}$. Define the feature matrix **X** to be an *n*-by-*m* matrix whose rows are the feature vectors of nodes. Thus, **X**_*ik*_ is the *k*-th feature of node *i*, and **X**_•*k*_, the *k*-th column vector of **X**, is the *k*-th feature of all nodes. In real-world cases, features usually contain noise.

In some typical link prediction methods (i.e. common neighbor method), nodes are assumed to be linked because they are similar. Now focusing on the *k*-th feature, we may measure to what degree dissimilar nodes are linked in the whole network by

$$\begin{array}{c} D_{k}^{\prime }=\sum_{i∼j}{\left(X_{ik}-X_{jk}\right)}^{2}=X_{•k}^{T}LX_{•k}\;, \end{array}$$

where *i* ∼ *j* indicates that *i* and *j* are neighbors, and **L** is the Laplacian matrix [41]. However, this measure is biased. In the rhs of the first equation, the feature **X**_*ik*_ of node *i* appears in *d*_*i*_ different terms in the summation, where *d*_*i*_ is the degree of node *i*. So features of high-degree nodes dominate the value of $D_{k}^{\prime }$, while in many real-world networks, most nodes are of low degree [1]. Thus the value of $D_{k}^{\prime }$ does not properly count the similarity of the features from the majority.

The rightmost term in the above equation is the quadratic form of the Laplacian. To treat features from different nodes equally, a natural alternative is using the quadratic form of the normalised Laplacian matrix $\tilde{L}$ [41],

$$\begin{array}{c} D_{k}=X_{•k}^{T}\tilde{L}X_{•k}\;. \end{array}$$ (9)

The quadratic form of $\tilde{L}$ has similar interpretation of that of **L**, so larger *D*_*k*_ indicates to a larger extent, dissimilar nodes are linked together. Thus *D*_*k*_ can be used as a non-biased dissimilarity measure of the *k*-th feature.

In signal processing, to filter out noise, the signal is decomposed into a set of sine waves with different frequencies. For higher frequencies, the sine waves oscillate much more rapidly. Then the waves with frequencies that are considered within the band of noise are filtered out. In our case, the eigenvectors of the normalised Laplacian provide a similar notion of frequency. To understand this, denote by *λ*_1_ < *λ*_2_ < ⋯ < *λ*_*n*_ the eigenvalues of the normalised Laplacian matrix $\tilde{L}$, and **v**_1_, **v**_2_, ⋯, **v**_*n*_ the corresponding eigenvectors. The Courant-Fischer Theorem [42] tells us that

$$\begin{array}{c} v_{1}=\arg\min_{x:{∥x∥}_{2}=1}x^{T}\tilde{L}x\;, \end{array}$$ (10)

and

$$\begin{array}{c} v_{l}=\arg\min_{x:{∥x∥}_{2}=1,x⊥\text{span}\{{\text{v}}_{1},\ldots {\text{v}}_{l-1}\}}x^{T}\tilde{L}x \end{array}$$ (11)

So, if **X**_•*k*_ = **v**_1_, then *D*_*k*_ achieves its smallest, which indicates that **v**_1_ oscillates slowly among connected nodes (since *D*_*k*_ is a dissimilarity measure). The eigenvectors associated with larger eigenvalues oscillate more rapidly.

Similar to filtering noise in signal processing, we can project **X**_•*k*_ onto {**v**_*i*_}, and filter out the components with high “frequency”, i.e., the components on **v**_*i*_ with large subscript *i*, since we treat the existence of links connecting dissimilar nodes as noise. Denote the cut-off threshold by *t*, the noise-filtered **X**_•*k*_ reads

$$\begin{array}{c} {\hat{X}}_{•k}=\left(v_{1}^{T}X_{•k}\right)v_{1}+\left(v_{2}^{T}X_{•k}\right)v_{2}+\cdots +\left(v_{t}^{T}X_{•k}\right)v_{t}=V_{t}V_{t}^{T}X_{•k}\;, \end{array}$$ (12)

in which **V**_*t*_ = [**v**_1_, **v**_2_, ⋯, **v**_*t*_] is a matrix whose columns are the first *t* eigenvectors of **L** with the smallest eigenvalues.

Since no prerequisite is required for *k*, we can easily generalise the above derivation for the *k*-th feature to any other feature. Then we obtain the noise-filtered features for the whole network

$$\begin{array}{c} \hat{X}=V_{t}V_{t}^{T}X\;. \end{array}$$ (13)

**Fig 1:**
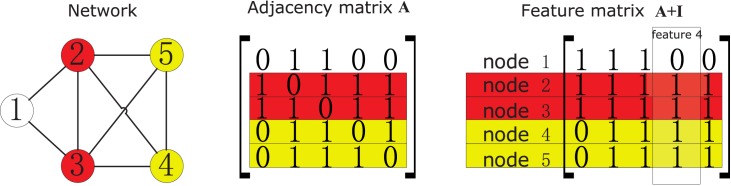
Demonstration of using rows of A + I as the feature vectors for nodes. In the network, nodes 4 and 5 are topologically equivalent. However, the 4th row of **A** reads [0, 1, 1, 0, 1], and the 5th reads [0, 1, 1, 1, 0], which are different. By adding **I**, the 4th and 5th rows of **A** + **I** now are both [0, 1, 1, 1, 1], which is exactly what we want. This is also the case for nodes 2 and 3. The *k*-th feature of a node can be interpreted as whether the distance between it and node *k* is no more than 1. For example, the distance between node 1 and 4 is greater than 1, while the distance between all the other nodes and node 4 are within 1, so the 4th feature is [0, 1, 1, 1, 1]^*T*^.

For any node *i*, its connections with all other nodes in the same network are totally characterized by the corresponding rows in the adjacency matrix **A**. So one may use these rows as the feature vectors for nodes, as in [43, 44], and interpret the *k*-th feature of node *i* as whether it is a neighbour of *k*. But there are some minor issues with this choice. Recall that the above derivation is based on the minimisation of the dissimilarity measure of all linked nodes (see Eq (9)). We now consider two linked nodes *i* and *j*, which have exactly the same neighbourhoods, so we expect the dissimilarity of them is 0. However, their *i*-th feature will not be the same, since the *i*-th feature of *i* is 0 while the *i*-th feature of *j* is 1. This is the same with the *j*-th feature. We can see from this analysis that one can use the rows of **A** + **I** rather than **A** as the feature vectors for nodes. So the *k*-th feature of node *i* can be interpreted as whether its to node *k* is no more than 1. This is further demonstrated in Fig 1.

Apply the above methodology, we have

$$\begin{array}{c} \hat{S}=V_{t}V_{t}^{T}\left(A+I\right)\;. \end{array}$$ (14)

Entries of $\hat{S}$ reflect the intrinsic connection pattern, so they can be used to predict missing links. However, since we are focusing on undirected networks, there is still one problem with $\hat{S}$. We can see that according to Eq (14), it might not be symmetric. So we will make predictions based on entries of $\frac{1}{2}\left(\hat{S}+{\hat{S}}^{T}\right)$ instead of $\hat{S}$.

### Experimental Results

To compare the performance of the Noise-Filtering (NF) method and some well-known algorithms, 12 real-world networks, including biological, social, and technological networks, are considered in the experiments. They are transformed into undirected, and simple (with multiple links or loops removed) networks. The resulting networks are summarized in Table 1.

**Fig 2:**
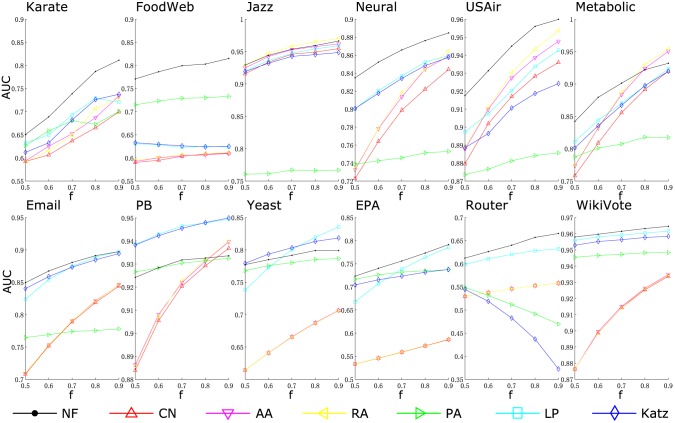
Comparison of prediction accuracy under the AUC metric. The fraction of training sets *f* is varied from 0.5 to 0.9.

**Fig 3:**
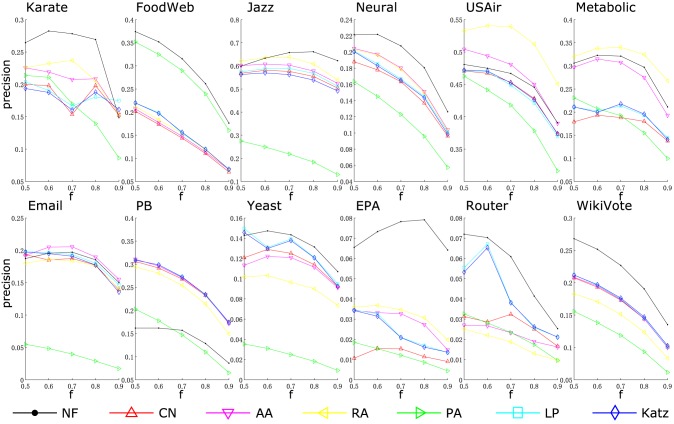
Comparison of prediction accuracy under the precision metric. The fraction of training sets *f* is varied from 0.5 to 0.9.

Table 2 shows the prediction accuracy measured by AUC. Results measured by another widely used metric, *precision*, is presented in Table 3. These metrics are introduced in the Methods section. The highest AUC/precision for each network (in each column) is shown in boldface. Under the AUC metric, NF performs best in 7 out of 12 networks, while under the precision metric, NF performs best in 9 of them. Figs 2 and 3 compare prediction accuracy of different algorithms under varied partitioning ratio. It can be seen that the proposed method is either the best or very close to the best, except for only one network—PB. Moreover, the robustness of the proposed method can also be verified by Figs 2 and 3. Since in most networks, the accuracy of the proposed method is either the best or very close to the best, even with the size of training sets varied.

Intuitively, the more the amount of known information, the higher the prediction accuracy. But in Fig 3, we see that most of the time, the precisions do not increase with the size of training sets. This is due to different sizes of probe sets (follow a conventional way, we always set *L* in Eq (2) to the size of the probe set). Thus with different sizes of training set, the precisions cannot be compared [45].

**Table 2:** Comparison of the prediction accuracy under the AUC metric in real-world networks.

	CN	AA	RA	PA	LP	Katz	NF
Karate	0.6994(162)	0.7338(202)	0.7281(182)	0.7006(297)	0.7206(200)	0.7375(284)	**0.8113(211)**
FoodWeb	0.6104(11)	0.6094(11)	0.6120(8)	0.7332(9)	0.6235(11)	0.6770(10)	**0.8150(8)**
Jazz	0.9545(2)	0.9619(2)	**0.9701(1)**	0.7668(8)	0.9591(1)	0.9485(2)	0.9663(1)
Neural	0.8441(4)	0.8589(4)	0.8644(4)	0.7529(7)	0.8595(6)	0.8575(5)	**0.8847(4)**
USAir	0.9359(3)	0.9477(3)	0.9537(2)	0.8856(5)	0.9427(3)	0.9242(3)	**0.9599(2)**
Metabolic	0.9198(3)	0.9506(2)	**0.9544(2)**	0.8172(7)	0.9233(3)	0.9195(4)	0.9319(2)
Email	0.8442(1)	0.8464(1)	0.8467(1)	0.7779(3)	**0.8974(1)**	0.8942(2)	0.8973(1)
PB	0.9368(0)	0.9396(0)	0.9398(0)	0.9325(0)	0.9495(0)	**0.9500(0)**	0.9336(1)
Yeast	0.7061(0)	0.7066(0)	0.7061(1)	0.7865(3)	**0.8357(1)**	0.8184(2)	0.7989(3)
EPA	0.5860(0)	0.5865(0)	0.5868(0)	0.7371(2)	0.7855(0)	0.7376(1)	**0.7915(2)**
Router	0.5580(0)	0.5579(0)	0.5579(0)	0.4694(3)	0.6320(0)	0.3738(3)	**0.6654(6)**
Wikivote	0.9337(0)	0.9347(0)	0.9344(0)	0.9484(0)	0.9616(0)	0.9584(0)	**0.9646(0)**

Each value is obtained by averaging over 100 implementations with independent random divisions of the training set(90%) and the probe set(10%). The method proposed in this paper is in the last column, NF (Noise Filtering). The best result achieved for each network data is in boldface. The numbers in the brackets denote the standard deviations. For example, 0.6994(162) means that the AUC value is 0.6994 and the standard deviation is 162 × 10^−4^.

**Table 3:** Comparison of the prediction accuracy under the precision metric in real-world networks.

	CN	AA	RA	PA	LP	Katz	NF
Karate	0.1525(96)	0.1538(156)	0.1538(146)	0.0863(68)	**0.1750(100)**	0.1613(123)	0.1487(93)
FoodWeb	0.0707(2)	0.0755(2)	0.0754(3)	0.1607(4)	0.0758(2)	0.1023(3)	**0.1762(5)**
Jazz	0.5044(6)	0.5244(6)	0.5393(5)	0.1300(4)	0.5120(7)	0.4920(6)	**0.6225(5)**
Neural	0.0962(2)	0.1039(3)	0.1025(3)	0.0575(2)	0.0985(3)	0.1027(2)	**0.1262(3)**
USAir	0.3730(8)	0.3898(8)	**0.4505(9)**	0.3164(7)	0.3738(9)	0.3695(8)	0.3905(9)
Metabolic	0.1378(4)	0.1932(4)	0.2680(5)	0.0999(4)	0.1449(5)	0.1408(4)	**0.2113(6)**
Email	0.1392(2)	0.1552(2)	0.1400(2)	0.0174(0)	0.1469(1)	0.1355(2)	**0.1503(2)**
PB	0.1729(0)	0.1716(0)	0.1493(0)	0.0652(0)	0.1735(0)	**0.1744(0)**	0.0861(11)
Yeast	0.0924(0)	0.0912(0)	0.0736(0)	0.0093(0)	0.0950(1)	0.0925(0)	**0.1070(1)**
EPA	0.0090(0)	0.0148(0)	0.0198(0)	0.0044(0)	0.0135(0)	0.0136(0)	**0.0642(0)**
Router	0.0166(0)	0.0162(0)	0.0096(0)	0.0096(0)	0.0212(0)	0.0226(0)	**0.0253(0)**
Wikivote	0.1009(0)	0.0999(0)	0.0833(0)	0.0616(0)	0.1005(0)	0.1028(0)	**0.1352(0)**

Each value is obtained by averaging over 100 implementations with independent random divisions of the training set(90%) and the probe set(10%). The method proposed in this paper is in the last column, NF (Noise Filtering). The best result achieved for each network data is in boldface. The numbers in the brackets denote the standard deviations. For example, 0.1525(96) means that the precision value is 0.1525 and the standard deviation is 96 × 10^−4^.

**Fig 4:**
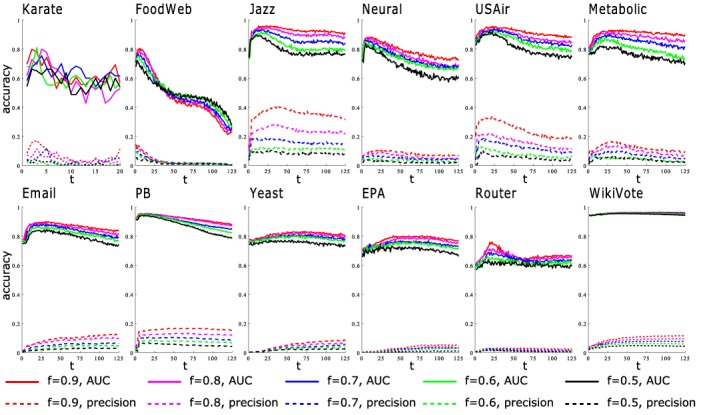
Prediction accuracy with different cutoff threshold *t* in the proposed noise-filtering method. The symbol *f* denotes the fraction of links in the training sets.

For all the parameter dependent methods considered in the experiment, i.e., LP, Katz, and NF, the results correspond to the optimal parameter, subject to the highest prediction accuracy. The optimal parameter can be found through a process similar to the *K*-fold validation. For example, in the proposed method NF, the training set is first partitioned into *K* units, a single unit is retained as the validation data for testing the method with specific *t*, and the remaining *K* − 1 units are used as known information. The cross-validation is then repeated *K* times (the folds), with each of the *K* units used exactly once as the validation data. The *K* results from the folds are then averaged. This whole process is repeated several times to find the optimal value of *t* (the value of optimal *t* is manually bounded in the range [1, 125], so the computation complexity is relatively small). In Fig 4, we see that for the two metrics considered here, the optimal *t* is robust, since the value of *t* where the prediction accuracy peaks does not change with the choice of the size of the training set. So there is no need to search for an optimal *t* in every single run of the simulation. Once the optimal *t* is found, it is set to this same value in all subsequent simulations, even with the size of the training set varied.

The experiments are conducted on a workstation with 64 GB RAM and an Intel (R) Xeon (R) E5-2687W @ 3.10 GHz 8-core processor. The comparison of computational time is summarized in Table 4. We see that the proposed method NF has similar run time with the global index Katz, especially on large networks, but having better performance.

**Table 4:** Comparison of the computational efficiency in real-world networks.

	CN	AA	RA	PA	LP	Katz	NF
Karate	0.2722	0.0863	0.0794	0.0741	0.0765	0.0767	0.3953
FoodWeb	0.1265	0.1332	0.1319	0.1408	0.1511	0.1659	0.2652
Jazz	0.1928	0.1939	0.2207	0.2117	0.2357	0.2596	0.4017
Neural	0.2041	0.2295	0.2302	0.2499	0.2491	0.3566	0.4580
USAir	0.2428	0.2789	0.2220	0.2788	0.3358	0.3118	0.5731
Metabolic	0.3635	0.3644	0.3881	0.4841	0.5499	0.5712	0.6719
Email	1.3969	1.6700	1.5221	3.2462	2.4013	5.0422	3.1099
PB	4.5587	5.0003	5.1569	6.0813	6.8293	9.7084	6.0244
Yeast	4.9859	6.8925	6.1101	13.9745	7.1093	21.4607	12.9680
EPA	20.3863	29.6148	26.7357	53.4191	24.4295	89.9699	50.5754
Router	19.2990	33.6029	25.6877	64.5513	23.8481	72.9081	89.1175
WikiVote	366.9862	387.7545	386.7545	447.2611	453.6359	704.4210	526.8122

Each value is the total time (in seconds) for 100 runs, with independent random divisions of the training set(90%) and the probe set(10%). The method proposed in this paper is in the last column, NF (Noise Filtering).

## Discussion

Real-world information always contains noise. This is also the case when making observation of a network structure. This problem is rarely considered in existing link prediction methods. To address this issue, we treat the connection of a given network as known information, and filter out the noises in it, based on an assumption that connected nodes should have similar neighbourhoods. The underlying regularity of the connection information is then retrieved and used to predict missing or future links. Experimental results show that it performs better than typical algorithms. Future works include how to improve the performance of existing methods based on the same idea of noise filtering.
